# Identification of Plausible Candidates in Prostate Cancer Using Integrated Machine Learning Approaches

**DOI:** 10.2174/0113892029240239231109082805

**Published:** 2023-12-20

**Authors:** Bhumandeep Kour, Nidhi Shukla, Harshita Bhargava, Devendra Sharma, Amita Sharma, Anjuvan Singh, Jayaraman Valadi, Trilok Chand Sadasukhi, Sugunakar Vuree, Prashanth Suravajhala

**Affiliations:** 1 Department of Biotechnology, Lovely Professional University, Jalandhar, Punjab, India;; 2 Bioclues.org, India;; 3 Department of Biotechnology and Bioinformatics, Birla Institute of Scientific Research, Jaipur, Rajasthan, India;; 4 Department of Computer Science, IIS University, Jaipur, Rajasthan, India;; 5 Urology and Renal Transplant Department of Renal Sciences, Rukmani Birla Hospital, Jaipur, Rajasthan, India;; 6 Department of Biotechnology, School of Bioengineering and Biosciences, Lovely Professional University, Punjab, Phagwara, 144001, India;; 7 Department of Computer Science, FLAME University, Pune, Maharashtra, India;; 8 Department of Urology and Renal Transplant, Mahatma Gandhi University of Medical Sciences and Technology, Jaipur, Rajasthan, India;; 9 MNR Foundation for Research & Innovation, MNR Medical College and Hospital, MNR University, Telangana, India;; 10 Amrita School of Biotechnology, Amrita Vishwa Vidyapeetham, Kollam, Kerala, India

**Keywords:** cBioPortal, prostate cancer, biomarkers, machine learning, bioinformatics, Prostate-Specific Antigens (PSA)

## Abstract

**Background:**

Currently, prostate-specific antigen (PSA) is commonly used as a prostate cancer (PCa) biomarker. PSA is linked to some factors that frequently lead to erroneous positive results or even needless biopsies of elderly people.

**Objectives:**

In this pilot study, we undermined the potential genes and mutations from several databases and checked whether or not any putative prognostic biomarkers are central to the annotation. The aim of the study was to develop a risk prediction model that could help in clinical decision-making.

**Methods:**

An extensive literature review was conducted, and clinical parameters for related comorbidities, such as diabetes, obesity, as well as PCa, were collected. Such parameters were chosen with the understanding that variations in their threshold values could hasten the complicated process of carcinogenesis, more particularly PCa. The gathered data was converted to semi-binary data (-1, -0.5, 0, 0.5, and 1), on which machine learning (ML) methods were applied. First, we cross-checked various publicly available datasets, some published RNA-seq datasets, and our whole-exome sequencing data to find common role players in PCa, diabetes, and obesity. To narrow down their common interacting partners, interactome networks were analysed using GeneMANIA and visualised using Cytoscape, and later cBioportal was used (to compare expression level based on Z scored values) wherein various types of mutation w.r.t their expression and mRNA expression (RNA seq FPKM) plots are available. The GEPIA 2 tool was used to compare the expression of resulting similarities between the normal tissue and TCGA databases of PCa. Later, top-ranking genes were chosen to demonstrate striking clustering coefficients using the Cytoscape-cytoHubba module, and GEPIA 2 was applied again to ascertain survival plots.

**Results:**

Comparing various publicly available datasets, it was found that BLM is a frequent player in all three diseases, whereas comparing publicly available datasets, GWAS datasets, and published sequencing findings, SPFTPC and PPIMB were found to be the most common. With the assistance of GeneMANIA, TMPO and FOXP1 were found as common interacting partners, and they were also seen participating with BLM.

**Conclusion:**

A probabilistic machine learning model was achieved to identify key candidates between diabetes, obesity, and PCa. This, we believe, would herald precision scale modeling for easy prognosis.

## 1 INTRODUCTION

Prostate cancer (PCa) has become the second most common cancer type in men and the fifth major reason for mortality across the world. As PCa is largely caused by the enlargement of the prostate gland with late onset, the difference in incidence rates in different parts of the world is due to the use of varying diagnostic approaches [1]. One in every nine men above the age of 65 is affected by PCa, which has become the most common cancer type in American men [2]. Around 1.6 million men are positively diagnosed every year, out of which 366,000 die [3]. The prostate-specific antigen (PSA) test has shown an undesirable high false-positive rate, revealing a poor prognosis. Alternatively, it has been proposed to lower the PSA threshold to solve the current problem with PSA testing. However, reducing the PSA threshold increases the risk of diagnosing painless disease and unnecessary treatment [4]. Over the years, there have been some developments that have improved the PSA testing and diagnostic accuracy, *viz.* measuring PSA with different molecular configurations and the rate of increase in PSA. Total PSA (tPSA) refers to the sum of free PSA (unbound) and bound PSA (mainly complexing with α1antichymotrypsin). The increase in tPSA, depicted by the total PSA rate (tPSAV), has now gained great attention in diagnosis and prognosis [4]. PSA seems difficult to completely replace because of its least invasive properties and cost-effectiveness; nevertheless, there is an urgent requirement to supplement PSA with other such biomarkers that could enhance both its specificity and sensitivity during screening tests. Figuring out a panel of this type of diagnostic and prognostic-associated biomarkers could be considered ideal when working with PSA. Even though PCa has a lot of heterogeneity, it is not ideal for forming the screening basis based on a single antigen to provide an accurate analysis report for every patient [4].

Genome-Wide Association Studies (GWAS) serve as a resource providing complete genetic association data to compare and discover significant datasets by taking genes, gene regions, phenotypes, or any trait through GWAS central (http://www.gwascentral.org last accessed May 23^rd^, 2022). It gives more than 67 million P-values for 1600 above studies, making it the world’s biggest database of GWAS information at the summary level [5]. On the other hand, the Prostate Cancer Association Group investigating cancer-associated alterations in the genome (PRACTICAL) is an international consortium formed to get precise evaluations of risk related to variants evaluated in large numbers of both cases and controls [6]. The rationale behind using this meta-centric approach is that there is a dearth of screening candidate genes and mutations overlaying both tools. Therefore, as a mandate of our recently carved out cancer prostate consortium of India [7], we attempt to undermine the candidate genes and mutations from the myriad of databases and examine whether or not any candidate prognostic biomarkers are centric to the annotation.

## 2 MATERIALS AND METHODS

### 2.1 Datasets

**Fig. (1):**
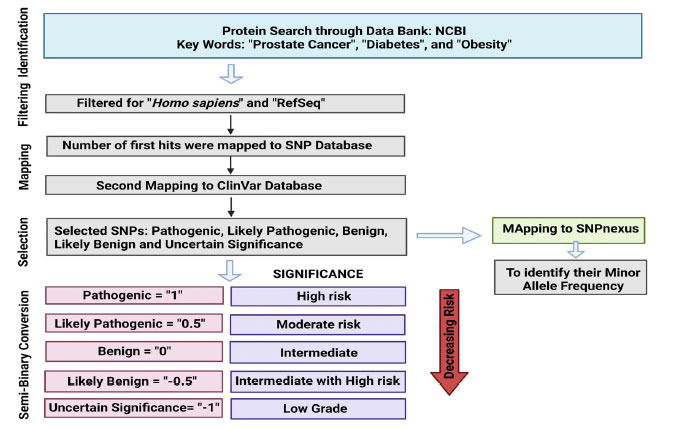
Flow chart representing identification, filtering, mapping (to SNP database, ClinVar, and SNPNexus), selecting, and finally, semi-binary data conversion (1, 0.5, 0, -0.5, -1) with respective significance. (Figure created through BioRender https://www.biorender.com/).

Separate catalogs of clinically verified variants (Clinvar) were first prepared for PCa, diabetes, and obesity from the NCBI with searches using keywords: *“Prostate Cancer”, “Diabetes”, “Obesity”, and* boolean expressions, *viz.* AND, OR, NOT were used wherever needed. The associated clinical parameters of comorbidities leading to cancer progression, especially for the prostate malignancy, were carefully chosen and were further categorized into unknown significance, likely benign, benign, likely pathogenic, and pathogenic, based on their threshold values with binary/semi-binary scores, *viz*. -1, -0.5, 0, 0.5, and 1, respectively. While these binary scores are given based on the risk of acquiring PCa that increases with the rising pathogenicity of a variant, the general stratification of low grade, intermediate but less risk, intermediate, moderate risk, and high risk corresponding with -1, -0.5, 0, 0.5, and 1, respectively, was checked and tabulated (Fig. **1** and Supplementary spreadsheet **1**). The data was then subjected to five different machine learning algorithms, namely linear regression, multilayer perceptron, random forest, random tree, and REP tree (Table **1**), and further compared to predict the chance of developing PCa when there are any change variables from the control population. Similarly, the annotated data of clinical attributes for all three diseases was transformed into semi-binary coding (-1, -0.5, 0, 0.5, 1) based on the different ranges and their corresponding degree of risk for developing PCa (Supplementary Fig. **1**).

### 2.2 Clinical Parameters

#### 2.2.1 Clinical Parameters of PCa

##### 2.2.1.1 Gleason Grading

Originally, Gleason grading was based on anatomical patterns seen in hematoxylin and eosin (H and E) stained sections of prostate adenocarcinoma instead of cellular characteristics. In that system, pattern 1 was regarded as a well-confined lump consisting of even, tightly packed, separate, well-distinguished, and moderate-sized glands, whereas pattern 2 showed several variations in neoplastic glands size with increased stroma in them and irregularities in their lump’s circumference. In pattern 3, some polyporus formed glands in gland structures called glomerulations, while fuse glands were seen in pattern 4. A blemish outgrowth demarcated it as pattern 5, where solid cord growth and tumor cell infiltration were seen later [8]. Modern biopsy approaches demanded more advances in Gleason grading to interpret and score biopsies. Hence, on the new grading system, Gleason's score less than or equal to 6 is categorized under grade group 1, a score of 3+4=7 under group 2, a score of 4+3=7 under group 3, a score of 4+4=8 under group 4, and a score of 9 -10 under grade 5. This latest advanced grading system is incorporated as a new addition to the World Health Organization classification for prostate tumors [8]. Scoring is the sum of highly frequent common and secondary patterns 3+5 [9]. As per current developments in immunohistochemistry, grades 1, 2, and 3 are almost identified as similar, hence are not much considered. However, in grade five, an interface between grade 3 or 4 and 6 or 7 is marginal between low risk and high risk of cancer [10].

##### 2.2.1.2 Prostate-Specific Antigen (PSA)

PSA is used in PCa screening at earlier stages to reduce the overall mortality rate but specific mortality and improve treatment approaches. However, PSA screening has still not shown remarkable results in saving patient lives. Besides this, PSA screening is accompanied by several problems, like overdiagnosis (false positive or false negative), which can lead to prolonged side effects of treatments [4].

##### 2.2.1.3 Digital Rectal Examination (DRE)

Digital rectal examination was largely used for earlier diagnosis of PCa before the advent of PSA. However, DRE can only detect several tumors because of its inefficacy in correlating location nodules with tumor locations in biopsy results. Therefore, these days, DRE tests are less recommended in routine PCa screening [11].

#### 2.2.2 Clinical Parameters of Diabetes Associated with Cancer/PCa

##### 2.2.2.1 Glycated Hemoglobin (HbA1c)

Glycated hemoglobin (HbA1c) testing is considered a gold standard for evaluating glycemic control in diabetic patients. It gives the average estimation of plasma glucose [12, 13]. A high level of HbA1c is interlinked with a chance of having hepatocellular carcinoma (HCC) among pre-existing diabetic patients. With every rise of 1% HbA1c level, the possibility of having HCC elevates by 26-50%. In insulin resistance (IR) in T2DM patients, because of prolonged use of antidiabetic therapies, exposure to free circulating insulin increases, and cellular mitosis gets stimulated by the insulin growth factor (IGF-1) intracellular pathway, a key mitogenic and antiapoptotic trigger in cancer development [14]. A threshold value of 6.1% is the optimum sensitivity and specificity, and 6.5% is the finest specificity to diagnose diabetes, as indexed in American Diabetes Association (ADA) recommendations [15, 16]. Its limitations include its association with poor performance in pregnant females, old age, and the chance of overdoing in anemia and genetically predisposed ones [17]. A study under UK Biobank has also suggested that high HbA1c is associated with several types of cancer with increased risk for stomach, liver, colon, bladder, esophagus, lungs, endometrium, pancreas, and kidneys and decreased risk for PCa, suggesting that diabetes and glycemic control is crucial in limiting cancer risk [17].

##### 2.2.2.2 WBC Count Test

WBC count testing alone can predict diabetes even in non-glycemic men. It has been estimated that for every 1000 cells/mm^3^ rise within the normal range, the chance of diabetes rises by 7.6%. In addition, chronic inflammation increases the likelihood of diabetes even without obesity in autoimmune-ailed patients. So, WBC count is considered an independent risk factor for diabetes in young people [18, 19]. A high WBC count is associated with an increased risk of venous thromboembolism (VTE) (arterial thrombosis and pulmonary embolism) in cancer patients. These cancer patients who developed VTE showed a short life span compared to those who did not develop VTE [20].

##### 2.2.2.3 Fasting Blood Glucose

Fasting or exposure to a nutrient-deprived (fast mimicking diet, FMD) environment of cancer cells brings alteration in growth factors and metabolites, which could lower the tendency of cancer to adapt and survive. This can be a possible way of refining the cancer treatment approaches [21]. Some epidemiological studies have presented that T2DM has an inconsistent effect on the risk of PCa at different points in time. It was suggested in some cases that, over time, diabetes has shown a protective impact on PCa development because of poor serum levels or less availability and activity of IGF-1 in the late stages of T2DM [22]. Fasting blood sugar testing is more reliable than HbA1c [23].

##### 2.2.2.4 Body Mass Index (BMI)

Overweight or obesity in adults has shown considerable chances of acquiring diabetes in a lifetime. However, with aging, its impact on the risk of diabetes, life span, and period of diabetes will weaken. Adults are affected and have a higher chance of mortality due to diabetes if the BMI level is above or equal to 30 kg per meter square [23]. A number of genetic variants are found common in GWAS, which confirms their associations [24].

#### 2.2.3 Clinical Parameters of Obesity Associated with Cancer/PCa

##### 2.2.3.1 BMI (Body Mass Index)

Accumulation of adipose tissue in excess amounts as a result of a high intake of calories as compared to the energy expenditure of the body is considered obesity [25]. It is quite evident that along with the risk of T2DM and cardiovascular diseases, several cancer types’ risk is also directly proportional to increasing body weight. Their interlinking can be explained based on altered endogenous hormone metabolisms like insulin, IGF and steroids, which deviate from living processes, cell proliferation, differentiation, and apoptosis from the normal equilibrium. Hence, checking on weight gain could significantly help in lowering cancer risk. A BMI of 18.5-25 kg/m^2^ is suggested to escape from this risk, even as, in some studies, it is found that there is a high risk of cancer even in the range of 20 to 25 kg/m^2^. Therefore, it has been highly advised to maintain weight in lower fields only [26]. A systemic pro-inflammatory environment caused by abdominal adiposity might initiate diabetes and cancer [27] as internal metabolic alterations in combination with several environmental factors trigger various other processes in the body that are required in the initiation of tumor development [25, 28]. If there is a family history of PCa, there are more likely chances of having the same with increasing BMI. So, it has been indicated that BMI is one of the clinical factors that could predict PCa during biopsies [29]. This is supported by a study clarifying that a higher BMI results in more mortality and moderate to high short-duration annual changes in BMI linked with less mortality rate in any cancer type [30].

##### 2.2.3.2 LDL/HDL

Several types of cancers, including aggressive PCa, are known to be caused because of obesity. Cholesterol is a known precursor of androgens, which plays a key role in PCa development. Cholesterol-related comorbidity called hypercholesterolemia, in association with obesity, is a promoter of both tumor proliferation and inflammation. Serum cholesterol is related to PSA and results in a high rate of PSA-based biopsies and diagnosis, resulting in high cholesterol in men. It can be concluded that high total serum cholesterol or HDL (high-density lipoprotein) is a risk factor for having a more aggressive form of PCa [31].

### 2.3 Machine Learning Algorithms

We have used the Waikato Environment for Knowledge Analysis (Weka) for implementing the machine learning algorithms. The algorithms used for the study included Linear regression, Multilayer perceptron, Random forest, Random tree, and REPTree for the regression analysis.

The annotated PCa, diabetes, and obesity data sets consist of three attributes: protein change, clinical significance (last reviewed), and semi-binary value to clinical relevance (Supplementary spreadsheet **2**). The first two attributes form the independent variables, while the third denotes the dependent variable. We evaluated the following regression algorithms (with the default set of parameters) on each of the PCa, diabetes, and obesity datasets using a train test split of 70:30. Based on the RMSE values and the average, the standard deviation (PCa=0.000612372; DM=0.012077094; Obesity=0.00128582 (Table **2**) and then the normalized deviation values are calculated and plotted against each other by line graphs.

### 2.4 Correlational Study

**Fig. (2):**
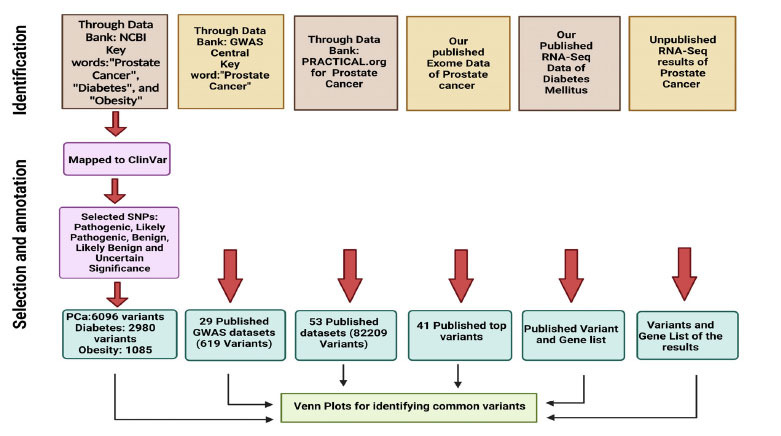
Flow chart for the correlational study of ClinVar variants (PCa, diabetes, and obesity) and published GWAS data from GWAS Central, PRACTICAL consortium, exome data, and RNA Seq data of both prostate cancer and diabetes mellitus for identifying common variants. (Figure created through BioRender https://www.biorender.com/).

To categorize this, the risk variants were retrieved from ClinVar, published GWAS data (from the PRACTICAL consortium and GWAS central), our own Exome data (Saxena *et al*., 2021), and RNA seq Diabetes Type 2 Mellitus data [32] (Fig. **2**). A cross-check was performed to identify common key players using Venn plots (https://bioinfogp.cnb.csic.es/tools/venny/).

### 2.5 Interactome Network

For visualizing the interaction network of the commonalities to find associations through their interacting partners, GeneMania is used wherein different types of gene-gene interactions by providing a seed list of our interest, which is then extended to incorporate other genes as interacting partners predicted to share the same function based on their overlapping connections in biological pathways [33].

### 2.6 CBioportal

We used datasets provided by Armenia *et al.* (2018) [34], wherein they identified 97 significantly mutated genes (SMGs), 70 of which were not earlier involved in PCa, followed by several mutations that were seen in less than 3% of the cases and another study used was of TCGA, Cell 2015 [35].

### 2.7 Differential Analysis: GEPIA 2 (Gene Expression Profiling Interactive Analysis)

GEPIA 2 facilitates the comprehensive analysis and complex data mining tasks of expression datasets from TCGA (The Cancer Genome Atlas) and GTEx (Genotype-Tissue Expression) [36]. Box plots were analysed for studying transcription profiles of different cancers in humans and normal tissues using the datasets of TCGA and GTEx in the GEPIA tool. It is one of the important publicly available and personalized tools for functions like correlation, survival, profiling, plotting, analysis, dimension reductional or differential expression analysis, and detection of a similar gene [37].

### 2.8 Survival Analysis

For the survival analysis, we used GEPIA 2 again, wherein the log-rank below 0.05 (*p*<0.05) is referred to as significant [38]. Survival plots are formed with Kaplan-Meier (KM curve), and 2 curves are then compared with the log-rank test [39].

### 2.9 Cytoscape

For ranking nodes of a network on the basis of network features, a plugin called CytoHubba was added to Cytoscape. It provides a platform for several topological analyses in retrieving subnetworks from the whole protein-protein interaction network. A list of a few nodes was taken from the complete network, and computed topological features were saved as node attributes in the Cytoscape data structure. The clustering coefficient based on the nodal size and color of the node related to the degree [40] was performed with a PPI network constructed using the STRING database by giving a list of genes in the query, and then its visualization and subnetworks were formed by Cytoscape using the bottleneck method. Later, topological parameters were analysed with the help of a plug-in, *viz.* network analyzer. It calculates different topological features like connectivity, node number, connecting edges, clustering coefficient, average clustering coefficient, centralization, connectivity degree, *etc*. [41].

## 3 RESULTS AND DISCUSSIONS

### 3.1 Comparison of Vivid Datasets Yields Candidate Genes Common to Diabetes and PCa

**Fig. (3):**
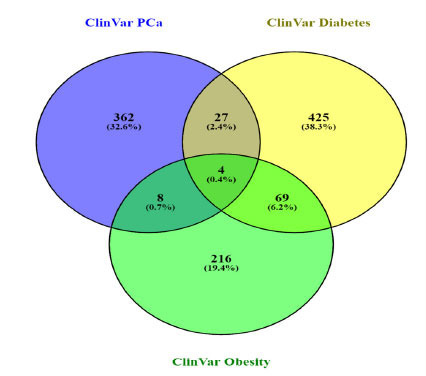
Venn plot for identifying commonalities between Clinvar datasets of prostate cancer, diabetes, and obesity. It showed 27 common genes between prostate cancer and diabetes, 8 common genes between prostate cancer and obesity, 69 common genes between diabetes and obesity, and 4 among all three diseases. *DNAJC6, SDHA, WRN, RET, MRE11, NF1, GNRHR, HSPB1, ACAT1, SHH, BIN1, STAT3, ZIC2, FBP1, PARK7, RGS2, SYNE2, ALDH6A1, BBOF1, HNF1B, AKT1, CACNA1H, KDM6B, KDM3B, HP, TXNL4B, BMP4, IRS1,* and *NC*L were common in PCa and diabetes. *BRCA2, FANCI, BAP1, AR,* and *PMS2* were common in PCa and obesity. *AHDC1*, *TOP3A*, *LEP*, *BBS12*, *FOXP3*, *BBS7, GATA6, GCK, POLG, GHRL, BBS1, SCN1A, BTK, CYP19A1, ASTN2, PDE4D, SHANK3, WDPCP, RPL36A-HNRNPH2, MKKS, IFT74, MC4R, BBS9, SLC12A3, GHRLOS, DMXL2, PAX6, TTC8, BBS4, NUDC, CEP19, NR0B2, IL1RN, BBS5, GLI2, TGIF1, LPL, MIR4713HG, ALMS1, UCP3, APC, ARL6, DARS2, LZTR1, SDCCAG8, PHKG2, MECP2, PROK2, NPHP1, SMARCB1, ATP7A, BBS10, ZDHHC24, APOA5, MPO, AVPR2, LOC106694316, DYRK1B, TMEM67, BBS2, CLCN5, GJB2, CORIN, ABCD1, ABCB4, TRIM32, MAGEL2, INSR, FOXP1, DDX3X,* and *APPL1* were common in diabetes and obesity with variants. *BLM,* TMEM67, RFX6, NR0B2, and NUDC were found to be common among PCa, diabetes mellitus, and obesity. (Figure created through Venny 2.1 https://bioinfogp.cnb.csic.es/tools/venny).

By comparing variants of all the individual comorbidities with PCa, we identified several common genes and variants along with some common protein changes. A few common variants were also identified in correlating different GWAS datasets with ClinVar data (Fig. **3**).

### 3.2 The GWAS Central Comparison Yielded rs721048 (*EHBP1*) and rs138213197 (*HOXB13*), which were found to be in ClinVar of PCa and GWAS Central

**Fig. (4):**
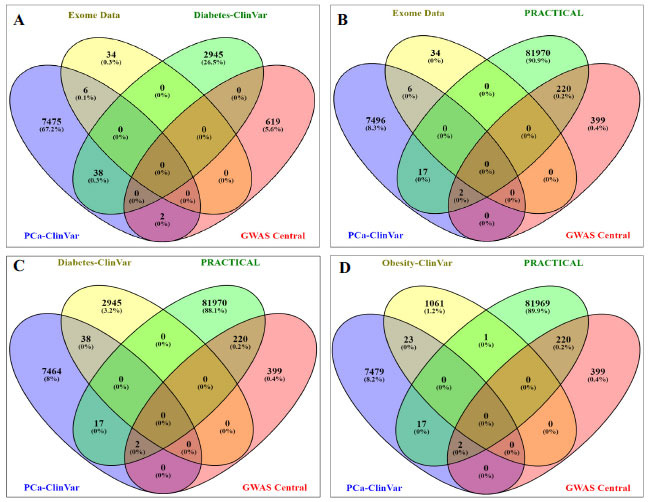
**A**, **B:** Venn plot to identify common variants among ClinVar prostate cancer, diabetes, GWAS central, PRACTICAL consortium for prostate cancer, and prostate cancer exome data; **C, D:** Venn plot to identify common variants among ClinVar prostate cancer, diabetes, obesity ClinVar, GWAS central, PRACTICAL consortium for prostate cancer and prostate cancer exome data. (Figure created through Venny 2.1 https://bioinfogp.cnb.csic.es/tools/venny).

**Fig. (5):**
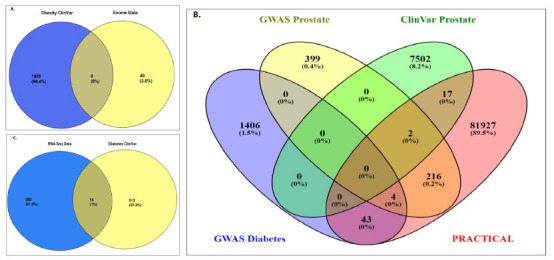
**A:** Venn plot showing no commonality between exome data and obesity ClinVar data; **B:** Venn plot for GWAS central data of both type 2 diabetes and prostate cancer, PRACTICAL Consortium, and ClinVar Prostate cancer; **C:** Venn plot showing some commonalities between RNAseq results of three prostate cancer samples and ClinVar data of diabetes. (Figure created through Venny 2.1 https://bioinfogp.cnb.csic.es/tools/venny).

In addition, 5 variants of *BRCA2* from exome data and PCa ClinVar data were found to be common, *i.e*., rs145988146, rs80358600, rs276174854, rs276174889, rs771203198, and 1 variant of *BRCA1* (rs28897696) GWAS central was common to both of them (Fig. **4**). Whereas the *PRACTICAL* consortium comparison yielded rs721048 (*EHBP1*) and rs138213197 (*HOXB13*), which were earlier found common to ClinVar PCa and GWAS Central, 17 other variants were also found common in the PRACTICAL consortium and ClinVar. Among them, 8 belongs to *BRCA1* (rs147297981, rs181430678, rs147509580, rs182524124, rs148500539, rs147856441, rs148068102, rs149141411), 2 of *FANCM (*rs147021911 and rs144567652), and 1 of each *MAD1L1* (rs121908982), *MSH6* (rs1800937), *PALB2* (rs45494092), *PNKP* (rs201872477), *XPC* (rs182616621), *POLD1* (rs149366027), and *EXOC8* (rs148264842) (Fig. **5B**). No commonality was observed between exome data and obesity (Fig. **5A**). One common variant, rs61816761, associated with the FLAG gene, was found to be common to obesity and diabetes.

### 3.3 RNA-Seq Data of both PCa and Diabetes Type 2 Mellitus

On comparing RNA-seq data of both PCa and DT2M with all the datasets, *PP1MB* and *SFTPC* were common in both types of RNA-seq and PCa ClinVar data (Fig. **5C**).

### 3.4 Interactome Networks Using GeneMANIA

**Fig. (6):**
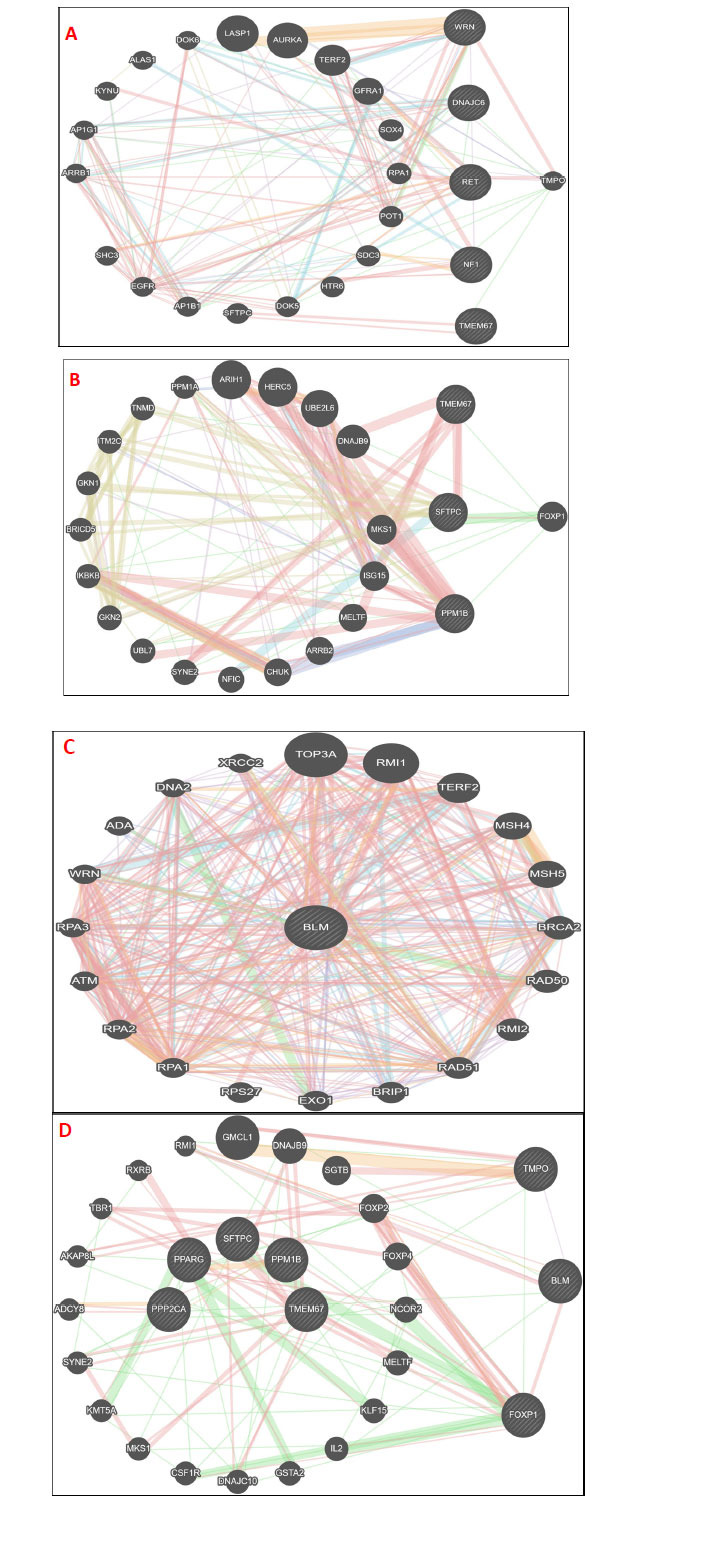
**A.** Interactome network of commonly associated genes (WRN, DNAJC6, RET, NF1AND TMEM67) of prostate cancer and diabetes from ClinVar data; TMPO is shown as a common interacting partner; **B.** Interactome network of TMEM67, SFTPC, and PPMIB showing mainly genetic interactions (green) with one common FOXP1; **C.** Interactome network of BLM gene found as the only one common gene among prostate cancer, diabetes, and obesity that interacted with both the earlier identified FOXP1 (physical interactions shown in red color) and TMPO (co-expression shown in purple color), which can be seen in **D**. Source: https://genemania.org

On applying GeneMANIA to the common genes of PCa and diabetes mellitus data of ClinVar, the interactome network showed a common interacting partner among them, which is *TMPO* (Fig. **6A**). The interactome network of *PP1MB* and *SFTPC* showed some commonalities in their interacting partners with other datasets. *SFTPC*’s interacting partner *TMEM67* is common in PCa ClinVar data and *FOXP1* in diabetes ClinVar data. *PPM1B*’s interacting partner PPP2CA is common in PCa ClinVar data, and *PPARG* is common in diabetes ClinVar. *TMEM67* is also common among the ClinVar data sets and *SFTPC,* whereas *FOXP1* was found as the common interacting partner of *PP1MB*, *SFTPC*, and *TMEM67* (Fig. **6**). *FOXP1* showed genetic interactions with both *SFTPC* and *PPM1B* (Fig. **6**). However, the rest of the interactome broadly showed physical interactions (red) with their functions and pathways involved enlisted from Genecards (www.genecards.org last accessed on May 25^th^, 2022) (Tables **3** and **4**). On the other hand, the BLM gene, which was found as the only common gene between prostate cancer and diabetes, interacts with both the earlier identified FOXP1 (physical interactions shown in red color) and TMPO (co-expression shown in purple color) (Fig. **6C** and **D**).

### 3.5 Machine Learning Result

The results can be further improved by including more independent variables with respect to each dataset. The linear regression and tree-based algorithms have a lower RMSE than the multilayer perceptron algorithm for the prostate cancer dataset. The linearq12 regression has the lowest RMSE as compared to multilayer perceptron and tree-based algorithms for the diabetes dataset. The tree-based algorithms have the lowest RMSE as compared to linear regression and multilayer perceptron algorithms for the obesity dataset.

### 3.6 cBioPortal Results

**Fig. (7):**
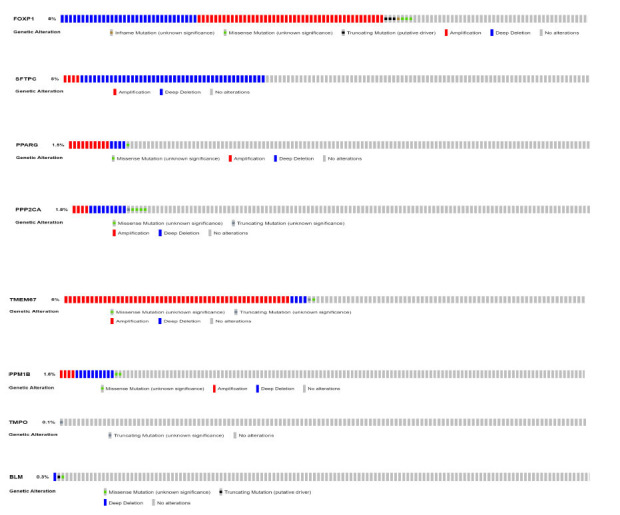
Overall percentage and type of genetic mutations in *FOXP1, SFTPC, PPP2CA, PPARG, PPM1B, TMEM67, TMPO*, and *BLM* related to prostate adenocarcinoma. (Source: www.cbioportal.org/)

On putting different queries of individual genes, it summarizes the genomic alterations across the whole sample, given the details about the frequency of gene mRNA (RNA-seq FPKM) related to its mutations from the selected study. Also, the graphical representation of protein domains and specific regions of a particular mutation in a gene is provided. Graphs show *FOXP1* with the highest 8% of alteration (amplification, deep deletions, in frame and missense mutation with unknown significance, and truncated mutations in putative driver), *SFTPC* with an overall 5% of alteration, which includes amplification and deep deletions (shown in blue), 1.8% in *PPP2CA* (amplification, deletions, missense mutation with unknown significance), PPARG with an overall 1.5% of alteration (amplification, deletions, missense mutation with unknown significance), *TMEM67* with overall 6% of alteration, which includes amplification, deep deletions, missense and truncated mutation with unknown significance, 1.5% of alteration (amplification, deletions, and missense shown in green) for *PPM1B* gene, *TMPO* with 0.1% of truncated mutation, and *BLM* with 0.3% of mutations (missense with unknown significance, truncation as putative driver and deep deletion) (Fig. **7**) in a prostate adenocarcinoma study by Abeshouse *et al.* (2018) [35].

### 3.7 Gene Expression Patterns were Viewed Using GEPIA 2

Using normal expression profile graphs (TPM) and box plots with the help of GEPIA 2, we performed a comparative expression analysis. Box plots were divided based on quartiles, with every box depicting the median range of expression of a particular gene in both normal and tumor samples separately. A horizontal bar in the middle of all boxes is the actual median of the expression, and both medians of tumor and normal are different. Outside the box, both below and above, a deviation limit is set; beyond that is known for outlier regions (abundant expression) (Fig. **8a**). Outliers in normal sample expressions might be a chance of experimental error or error in replicates. The prostate adenocarcinoma (PRAD) dataset was used to compare 492 tumors with 152 normal sample expression data, and later, the multigene expression comparison was rendered based on Z scores.

Comparing only tumor tissue expressions and matching TCGA normal and GTEx data, we found that the *PPP2CA* (5.5) and *FOXP1* (4.9) are highly expressing genes as compared to others, whereas TMEM67 and PPARG are low expressing, and *TMPO, SFTC,* and *PPM1B* are least expressing genes (Fig. **8b**). TMPO showed a very small change in expression profile from the normal but with a slightly higher deviation from the median expression in the tumor (Fig. **8c**), and the most outlied expression was seen for the *PPARG* gene (Fig. **8a**).

### 3.8 Top-ranking Genes Showed Vivid Clustering Coefficients for Ascertaining Survival Plots

**Fig. (9):**
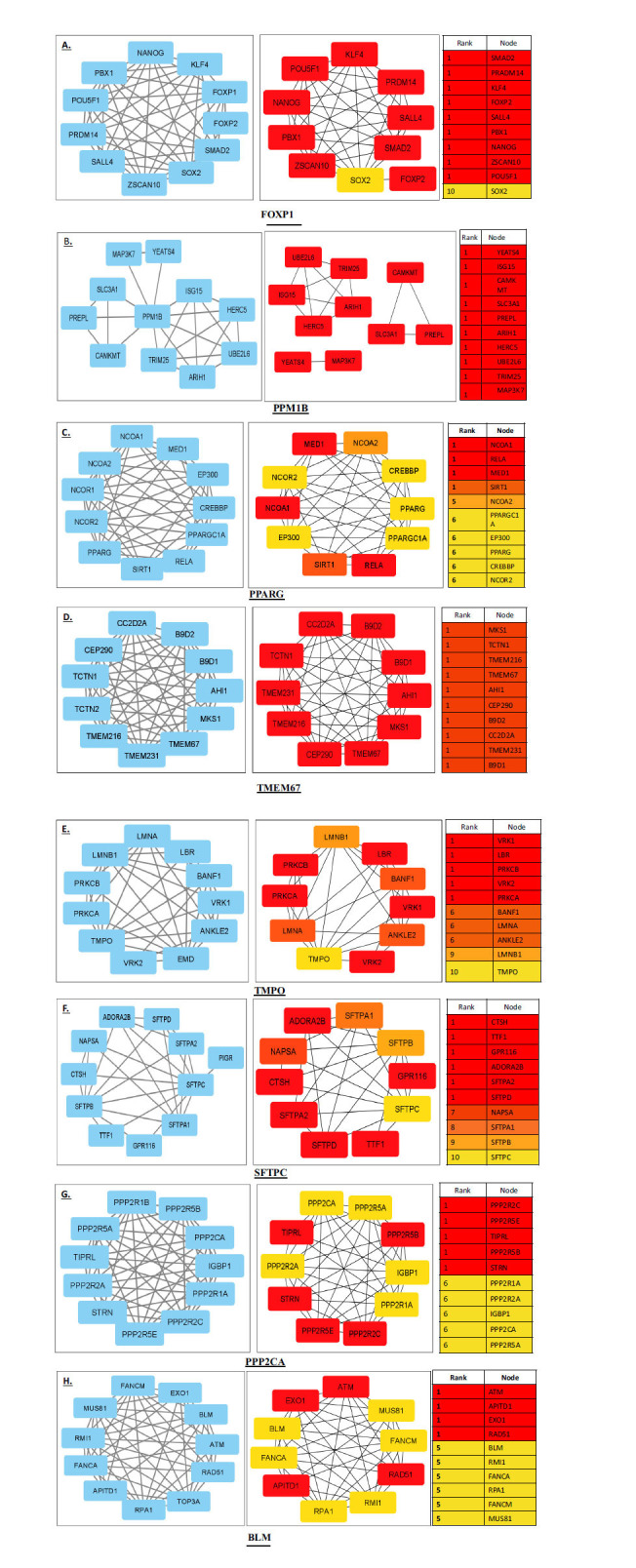
Clustering coefficient (CC) networks of all the identified genes (A. *FOXP1,* B. *PPM1B,* C. *PPARG,* D. *TMEM67,* E. *TMPO,* F. *SFTPC,* G. *PPP2CA* and H. BLM*)* formed by cytoHubba plugin in Cytoscape to segregate and analyse highly interacting gene groups that might be participating in same biological function. The degree of clustering coefficient is represented in these networks through a colour scale ranging from red to yellow. The highest (top-ranked) CC is shown in red, moderate are in orange, and least are in yellow (low rank). Source: Software Cytoscape.

The PPI visualization of all the major genes using Cytoscape-CytoHubba revealed a significant number of nodes and edges of the topmost stable and highest-scored genes in their respective networks based on the degree clustering coefficient (Fig. **9**).

**Fig. (10):**
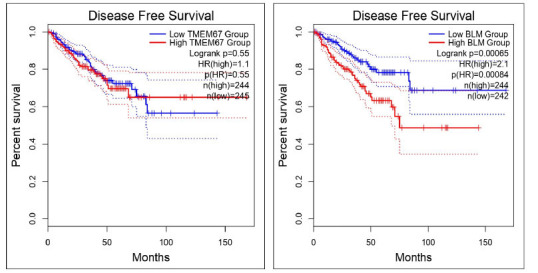
Comparison of overall survival (OS) and disease-free survival (DFS) of *FOXP1* and *BLM using* the GEPIA 2 tool with *PRAD* dataset for both TCGA normalized data and GTEx data. Source: http://gepia.cancer-pku.cn/

On analysing, we found that the disease-free survival for *FOXP1* is log-rank *p*=0.005), and *BLM* is log-rank with *p*=0.00065 (Fig. **10**). In a comparison of overall survival and disease-free survival in reference to the significance, only *BLM* was observed to have a significant p-value in disease-free survival. The insignificance with respect to other genes of *p-*value may be because of the individual genetic variability. Each study exhibits clinical heterogeneity. Therefore, we need to include higher sample size studies. Comparison of other genes’ overall survival (OS) and disease-free survival (DFS), *TMPO, TMEM67, TMPO, PPP2CA*, *PPM1B*, and *PPARG using* GEPIA 2 tool with *PRAD* dataset for both TCGA normalized data and GTEx data are shown in Supplementary Fig. (**2**).

Prostate cancer is age-linked and the second most common type of cancer among men. Most commonly, it is found in people above the age of 63 years. *PSA* is a widely used diagnostic biomarker with a high rate of false-positive results. Considering the heterogeneity of prostate cancer only, it does not seem justified to depend on a single biomarker for its screening and diagnosis for every patient. However, it seems difficult to replace such a biomarker as *PSA,* which has the least invasiveness and cost-effectiveness properties. A PRACTICAL consortium was established to get precise evaluations of risk related to different genetic variants and assessment of their combined associations of such variations by evaluating variants in large numbers of both cases and controls, and their GWAS datasets were published. Another resource called GWAS central (http://www.gwascentral.org) can provide complete genetic association data at a summary level, which is designed to serve its maximum utility and safe open approach. Our study categorically was divided into two parts, wherein the former part is the use of machine learning algorithms to predict the chance of developing prostate cancer, and the later part is completely the correlational study on both publically available datasets and published RNA-seq and WES results of prostate cancer and diabetes to identify the common associations among them, giving a *vice versa* relationship to help in early diagnosis of PCa. Later, different bioinformatic tools were used to study their interaction networks and their expression patterns.

For the risk prediction model, individual sets of clinically verified variants (Clinvar) were first prepared for PCa, diabetes, and obesity from the NCBI. The datasets were retrieved by using keywords, *viz. “Prostate Cancer”, “Diabetes”, and “Obesity”*. The data was later provided and labelled with binary/semi-binary scores (-1, -0.5, 0, 0.5, and 1) based on the clinical significance attributes as unknown significance, likely benign, benign, likely pathogenic, and pathogenic, considering the lowest to the highest risk ranges. The weak results predicted the chance of developing PCA when there are any changes in variables from the normal included in the machine learning. The prediction was done on the basis of the root mean square error standard deviation (PCa=0.000612372; DM=0.012077094; Obesity=0.00128582). As the data was normalized and plotted, we observed that the linear regression and tree-based algorithms were shown to have a lower RMSE than the multilayer perceptron algorithm for the PCa dataset. As the linear regression has the lowest RMSE compared to multilayer perceptron and tree-based algorithms for the diabetes dataset, the tree-based algorithms also have the lowest RMSE as compared to linear regression and multilayer perceptron algorithms for the obesity dataset. We argue that this prediction invariably would help in clinical decision-making.

## CONCLUSION

In 1980, prostatic acid phosphatase (PAP) was the first described biomarker of PCa progression. Its levels were found to be high in metastatic patients. Later, this was replaced by PSA, a serine protease secreted by epithelial cells inside the prostate gland, and its level was also found to be increased compared to normal in PCa patients [42]. Although the management and survival rate of this disease have been improved with PSA screening since 1980, its limitations have kept the study of more precise biomarkers for prostate cancer evolving fast. The recent genomic and proteomic technologies have helped to understand the biology of PCa in better ways to contribute to biomarker discovery [42]. The PSA's role is controversial in screening asymptomatic men because of overdiagnosis and consequent overtreatment without any lethal ailment. However, it is not only broadly used as a biomarker for screening but also in therapy response monitoring and risk stratification for relapse [43, 44]. Identification of potential biomarkers could certainly improve screening, diagnosis, and prognosis; therefore, apart from other biomarkers, isoform assays of PSA are more concentrated in current studies, and those biomarkers could predict aggressiveness and put forward better treatment approaches [44, 42]. As per the approval of the US Food and Drug Administration or Clinical Laboratory Improvement Amendments-based laboratory-developed tests, recent markers can be identified from urine and serum or can be tissue-based. Various tests include *TMPRSS2*-*ERG* gene fusion test, Mi-Prostate score test, Oncotype DX test, ProMark test, ConfirmMDx test, Prolaris test, Prostate Core Mitomic test, 4K score test, Prostarix test, Decipher test. Other include α-Methylacyl coenzyme A racemase (AMACR). *PTEN* gene deletions are not approved by the US FDA but are commercially developed and available as Clinical Laboratory Improvement Amendments-based laboratory-developed tests. Only a few have been approved yet by the US FDA, including *PSA, PHI,* and *PCA3*. Emerging biomarkers like tumor cells, microRNAs, and exosomes are still in the infant stage due to certain reasons like their flawed preclinical trials, inapt statistical analysis, *etc*. Hence, vigilant validation of each biomarker could only help to resolve prevailing or unmet challenges and lead to better diagnosis by clinicians, and the goal of personalized medicine could be achieved [42].

The ML heuristics has set a precedent to bring a transformation in cancer with data-driven pipelines for understanding possible causal relationships. What we sought to achieve through this pilot is to check whether candidate genes could be considered for precision scale modeling, and therefore, we employed prediction scale and analytics. The analysis revealed several common genetic factors shared among PCa, diabetes mellitus, and obesity. Specifically, *BLM*, *TMEM67*, *RFX6,* and *NUDC* found common genes among these conditions when comparing their data from ClinVar. A single variant, rs61816761, associated with the *FLAG* gene, was found common to both obesity and diabetes with GWAS Central data of PCa. Our RNA-seq data showed *PP1MB* and *SFTPC* as common in PCa and diabetes. By using GeneMANIA to commonalities, we obtained a network with common interacting partners between *TMPO* and *FOXP1*. While *FOXP1* was found to be a common interaction partner of *PP1MB*, *SFTPC* and *TMEM67,* an intriguing finding was that the BLM gene was the only common gene among PCa, diabetes mellitus, and obesity, interacting with both *FOXP1* and *TMPO*. The strength of our study lies in the comprehensive analysis of genetic factors in PCa, diabetes mellitus, and obesity, as well as its effective integration of diverse datasets, identification of common genetic variants, and the application of advanced analytical techniques. The current study employed a range of statistical and ML algorithms to analyse the data that identified common genetic factors and assessed predictive models. The lower RMSE values for specific algorithms in each dataset demonstrated the feasibility of using these methods for future research and clinical application. Nevertheless, our work has limitations, where we lack clinical interpretation as we set a hypothesis that the differentially expressed genes (DEGs) harbour certain mutations that tend to be pathogenic. To achieve this, we need a large number of datasets to be screened. Our CAPCI and Systems Genomics Lab are working towards these goals.

On a granular level, this analysis has allowed us to bring insights into ascertaining three different phenotypes in the form of diabetes, PCa, and obesity. There is room for better ML-based integration, and time will reveal where we stand.

## Figures and Tables

**Fig. (8a):**
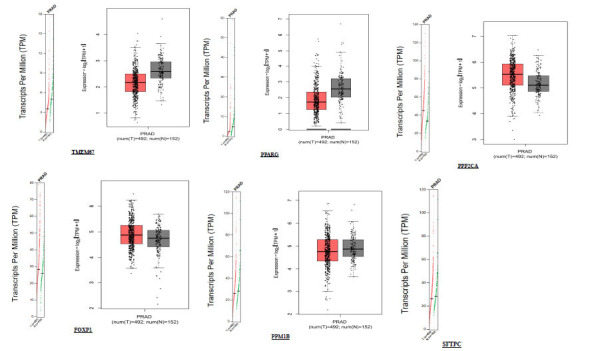
Box plots for expression of common genes compared to TCGA tumor-normal datasets of prostate adenocarcinomas (PRAD). Source: http://gepia.cancer-pku.cn/

**Fig. (8b):**
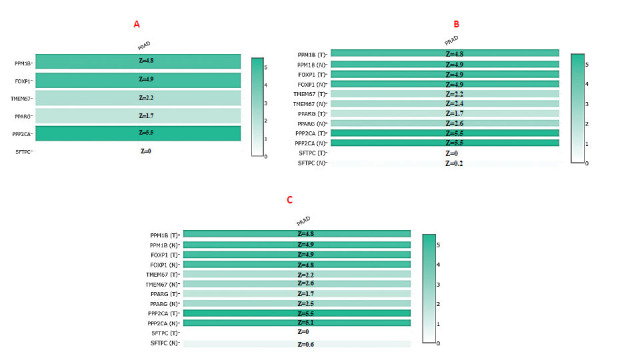
Box plots for expression of common genes in comparison to TCGA tumor-normal datasets of prostate adenocarcinomas (PRAD) and GTEx data (multiple genes based on Z scores) A. Only tumor tissue expression; B. Match TCGA normal data; C. Match TCGA normal and GTEx data. Source: http://gepia.cancer-pku.cn/

**Fig. (8c):**
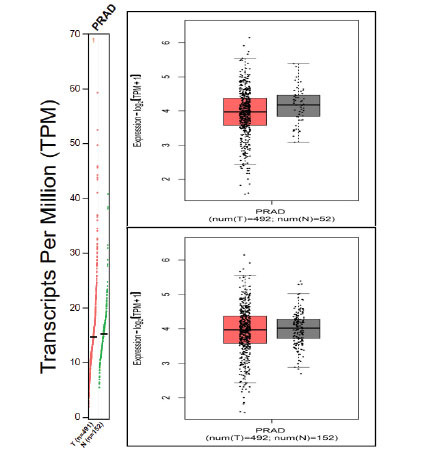
Expression in comparison to TCGA tumor-normal dataset of prostate adenocarcinomas (PRAD) dataset and GTEx data for TMPO gene. Source: http://gepia.cancer-pku.cn/

**Table 1:** List of different regression algorithms used for regression analysis.

**Algorithm**	**Details**
Linear regression	This algorithm is used to model a linear relationship between multiple independent variables and the target variable using a linear function. The main objective is to minimize the sum of squared errors between the original values and the predicted values of the target variable. This algorithm differs from simple linear regression in the way that the latter handles only one input feature while the former is capable of handling multiple input features.
Multilayer perceptron (MLP)	MLP is an artificial neural network that can be used both for classification and regression tasks. It is used to model the non-linear relationships between the input features and the output/target variable. A typical MLP network contains an input layer, an output layer, and a number of intermediate hidden layers. With the Weka tool, one can define several configurations of the network while specifying the activation function, number of hidden layers, and number of nodes per hidden layer learning rate parameters.
Random forest	A random forest algorithm is an ensemble of decision trees, each trained on a random set of features and a random set of samples. This sampling procedure tends to minimize the overfitting and induces diversity in the ensemble. The random forest can be configured using WEKA in terms of the number of trees, the number of input features at each split, and tree depth parameters. It can also be used to model non-linear relationships between the input features and the target variable both for classification and regression tasks.
Random tree	This algorithm is used for both classification and regression tasks. It considers a set of decision trees, each constructed using a subset of input features. The predictions from the individual trees are aggregated to generate the final predictions. As for random forest, WEKA provides parameters to configure the results of the algorithm.
REPTree	REPTree (Reduced Error Pruning Tree) is a decision tree algorithm that works by partitioning the input data by considering the feature that produces the best split at each node of the tree. After building the tree, the algorithm applies reduced error pruning to discard the branches that do not improve the accuracy of the tree. This process involves removing each subtree of the tree while evaluating the performance of the pruned tree on a validation set. If the performance of the pruned tree is the same or better than the original tree on the validation set, the subtree is discarded.

**Table 2:** List of different regression algorithms applied in Weka software on datasets of PCa, diabetes mellitus, and obesity and their root mean square error (RMSE) value.

**Dataset Used**	**Algorithm**	**Root Mean Square Error (RMSE)**
Prostate cancer	Linear regression	0.5419
	Multilayer perceptron	0.5434
	Random forest	0.5419
	Random tree	0.5419
	REPTree	0.5419
Diabetes	Linear regression	0.5975
	Multilayer perceptron	0.5977
	Random forest	0.6022
	Random tree	0.6022
	REPTree	0.6022
Obesity	Linear regression	0.2982
	Multilayer perceptron	0.2985
	Random forest	0.2981
	Random tree	0.2981
	REPTree	0.2981

**Table 3:** Functions and pathways involved in the common genes among ClinVar PCa and ClinVar diabetes from GeneCards.

**Gene**	**Function**	**Pathways Involved**
*WRN*	Magnesium and ATP-dependent DNA multifunctional helicase enzyme, 3'->5' exonuclease (ds DNA) at 5’ overhangs. It plays key roles in joint DNA molecule dissociation, which might end up giving homologous recombination products, formation of focal centers while replication, and DS break repair after gamma-irradiation. It also increases DNA polymerase obstruction at DNA lesion sites.	● Homology directed repair ● Homologous DNA pairing and strand exchange ● Regulation of TP53 activity ● Resolution of D-Loop structures ● SUMOylation ● Cell cycle checkpoints ● Gene expression ● Metabolism of proteins ● DNA damage ● Cell cycle, mitotic ● Telomere C-strand (lagging strand) synthesis ● DNA damage_NHEJ mechan- isms of DSBs repair ● Regulation of telomerase
*DNAJC6*	It promotes the uncoating of clathrin-coated vesicles by recruiting HSPA8 or HSC70 and clathrin-mediated endocytosis in neurons.	● Clathrin-derived vesicle budding ● Vesicle-mediated transport ● Clathrin-mediated endocytosis
*RET*	It plays a role in neuronal navigation, cell proliferation, cell differentiation, and cell migration upon binding with glial cell-derived neurotrophic factor family ligands, PTK2/FAK1 phosphorylation, and regulates the balance between both cell death and survival. It is active without ligand and triggers apoptosis by a process that needs receptor intracellular caspase cleavage. It behaves as a dependence receptor in the presence of the ligand GDNF in somatotrophs, promotes survival, and downregulates growth hormone (GH) production, and if GDNF is absent, it triggers apoptosis.	● RET signaling ● Developmental biology ● Cytokine signaling in the immune system ● Innate immune system ● Signaling by GPCR ● Tyrosine kinases / Adaptors ● VEGF pathway (Tocris) ● G-protein signaling_H-RAS regulation pathway ● Aryl hydrocarbon receptor ● Dopaminergic neurogenesis ● Signaling events regulated by Ret tyrosine kinase ● Sudden Infant Death Syndrome (SIDS) susceptibility pathways
*NF1*	NF1 is known to stimulate the Ras GTPase activity. It might be regulating the activity of Ras, has more affinity for Ras GAP, and lessens its particular activity.	● MAP kinase signaling ● Ras signaling ● Endometrial cancer ● Development of VEGF signaling and activation ● Oncogenic MAPK signaling ● Integrated breast cancer pathway ● Prolactin signaling
*TMPO*	It encodes many different LEM domains comprising isoforms of proteins that are involved in gene expressions, replication, cell cycle control, and chromatin organization. Alpha isoform encoded by it is mostly diffused in the nucleus and has a lamin binding domain, whereas beta and gamma isoforms are located on nuclear membranes containing HDAC3 interaction domains.	● Cell cycle, Mitosis (M Phase) ● Nuclear envelope reassembly ● Transport of the SLBP- independent mature mRNA ● Mitotic metaphase and anaphase (depolymerization of the nuclear lamina) ● Apoptosis and autophagy

**Table 4:** Details of common genes retrieved using geneCards.

**Gene**	**Function (UniProtKB)**
*SFTPC*	Elevates alveolar stability by reducing surface tension at the air-liquid interface in the peripheral air spaces.
*PPM1B*	It encodes an enzyme that has a large specificity. This enzyme can dephosphorylate PRKAA1 and PRKAA2, CDK2 and CDK6 *in vitro*. Its dephosphorylation at 'Ser-17’in can inhibit TBK1-mediated antiviral signaling. It has an important role in terminating TNF-alpha-mediated NF-kappa-B activation by dephosphorylating and inactivating IKBKB/IKKB.
*PPP2CA*	Important phosphatase for microtubule-associated proteins (MAPs), modulates the phosphorylase B kinase casein kinase 2 activity, MAP-2 kinase, and mitogen-stimulated S6 kinase; protects centromeric cohesion in oocytes, especially during meiosis I; can dephosphorylate SV40 large T antigen as well as p53/Tp53; activation of RAF1 by dephosphorylating it at 'Ser-259'; dephosphorylation of WEE1, which prevents its ubiquitin-mediated proteolysis; increase levels of WEE1 protein; G2/M checkpoint promotion; dephosphorylation of MYC and its ubiquitin-mediated proteolysis; dephosphorylation of FOXO3 which promotes its stabilization.
*PPARG*	It is a nuclear receptor and binds peroxisome proliferators like fatty acids and hypolipidemic drugs; modulates the transcription of its target genes like acyl-CoA oxidase; important regulator of glucose homeostasis and adipocyte differentiation; critical regulator of gut homeostasis through NF-kappa-B-mediated proinflammatory responses suppression; regulates the transcription of ARNTL/BMAL1 in the blood vessels, which controls cardiovascular circadian rhythms.
*TMEM67*	Important for ciliary structure and function; may regulate ciliary membrane composition; during early ciliogenesis, it helps in centrosome migration to the apical cell surface; plays a role in maintaining cilia length and appropriate number through the control of centrosome duplication; needed for cell branching morphology; important in endoplasmic reticulum-associated degradation (ERAD) of surfactant protein C (SFTPC).
*FOXP1*	Transcriptional repressor, acts with CTBP1 and synergistically represses transcription; plays role in the specification and differentiation of lung epithelium; regulates fate of lung secretory epithelial cell and regeneration by restricting the goblet cell lineage program; important regulator of B-cell development in transcription; regulates proliferation of cardiac muscle cell; helps in columnar organization of spinal motor neurons and promoting both lateral motor neuron column (LMC) and preganglionic motor column (PGC) formation and is needed for appropriate motor axon projections; regulates PITX3 promoter activity; may aid in identity of midbrain in embryonic stem cell-derived dopamine neurons by regulating PITX3; down-regulates T follicular helper cells T(FH)s differentiation; maintains hair follicle stem cell quiescence; represses several pro-apoptotic genes transcription and work together with NF-kappa B-signaling to promote B-cell expansion through inhibition of caspase-dependent apoptosis.

## Data Availability

The authors confirm that the data supporting the findings of this research are available within the article.

## LIST OF ABBREVIATIONS

ADA: American Diabetes Association
DEGs: Differentially Expressed Genes
DFS: Disease-free Survival
GTEx: Genotype-Tissue Expression
GWAS: Genome-Wide Association Studies
HCC: Hepatocellular Carcinoma
HDL: High-density Lipoprotein
IR: Insulin Resistance
ML: Machine Learning
OS: Overall Survival
PAP: Prostatic Acid Phosphatase
PCa: Prostate Cancer
PRAD: Prostate Adenocarcinoma
PSA: Prostate-specific Antigen
SMGs: Significantly Mutated Genes
TCGA: The Cancer Genome Atlas
VTE: Venous Thromboembolism
