# Supplementary material for: Identification of Plausible Candidates in Prostate Cancer Using Integrated Machine Learning Approaches
Source: Curr Genomics. 2023 Dec 20;24(5):287–306. doi: 10.2174/0113892029240239231109082805 (PMC10790336; doi:10.2174/0113892029240239231109082805)
Supplement: Supplementary file 1 [file CG-24-287_SD1.pdf]

Supplementary Material

Identification of Plausible Candidates in Prostate Cancer Using Integrated Machine Learning Approaches

Bhumandeep Kour[1,2,#], Nidhi Shukla[2,3,#], Harshita Bhargava[4,#], Devendra Sharma[5], Amita Sharma[4], Anjuvan Singh[6], Jayaraman Valadi[7], Trilok Chand Sadasukhi[8], Sugunakar Vuree[2,9,*] and Prashanth Suravajhala[2,10,*]

[1]Department of Biotechnology, Lovely Professional University, Jalandhar, Punjab, India; [2]Bioclues.org, India; [3]Department of Biotechnology and Bioinformatics, Birla Institute of Scientific Research, Jaipur, Rajasthan, India; [4]Department of Computer Science, IIS University, Jaipur, Rajasthan, India; [5]Urology and Renal Transplant Department of Renal Sciences, Rukmani Birla Hospital, Jaipur, Rajasthan, India; [6]Department of Biotechnology, School of Bioengineering and Biosciences, Lovely Professional University, Punjab, Phagwara, 144001, India; [7]Department of Computer Science, FLAME University, Pune, Maharashtra, India; [8]Department of Urology and Renal Transplant, Mahatma Gandhi University of Medical Sciences and Technology, Jaipur, Rajasthan, India; [9]MNR Foundation for Research & Innovation, MNR Medical College and Hospital, MNR University, Telangana, India; [10]Amrita School of Biotechnology, Amrita Vishwa Vidyapeetham, Kollam, Kerala, India

A. Clinical parameters of Prostate cancer

Clinical Significances of a Gene	Binary Scoring
Uncertain Significance	-1
Likely Benign	-0.5
Benign	0
Likely Pathogenic	0.5
Pathogenic	1

a. Clinical Significance

Threshold Values	Significance	Binary Scoring
0-2.5ng/ml	Safe	-1
2.6-4ng/ml	Safe but risky	-0.5
4-10ng/ml	Suspicious/Low risk	0
10-20ng/ml	Intermediate Risk	0.5
above 20ng/ml	High risk	1

b. PSA level

Grading Scores (0-10)	Staging	Binary Score
Group1 (0-6)	Low grade	-1
Group2 (3+4=7)	Intermediate with less risk	-0.5
Group3 (4+3=7)	Intermediate with more favourable	0
Group 4 (8)	Moderate risk	0.5
Group5 (9-10)	High risk	1

c. Gleason Grading groups

Clinical Significances of a Gene	Frequency Threshold values	Binary Score
Uncertain Significance	-	-
Likely Benign	-	-
Benign	-	-
Likely Pathogenic	>0.05	0
Pathogenic	<0.05	1

d. Minor allele frequency

DRE Physical Inferences	Binary Score
-	-
-	-
Only one side	0.5
Both sides	0
Spread beyond prostate	1

e. DRE

B. Clinical parameters of diabetes associated with Cancer/Prostate cancer

Clinical Significances of a Gene	Binary Scoring
Uncertain Significance	-1
Likely Benign	-0.5
Benign	0
Likely Pathogenic	0.5
Pathogenic	1

a. Clinical Significance

Clinical Significances of a Gene	Frequency Threshold values	Binary Score
Uncertain Significance	-	-
Likely Benign	-	-
Benign	-	-
Likely Pathogenic	>0.05	0
Pathogenic	<0.05	1

b. Minor allele frequency

Concentration Level	Significance	Binary Score
-	-	-
-	-	-
≥5.7, <6.5	Prediabetes (Likely Benign)	0
≥6.5%	Diabetes (Benign)	1

c. Glycated hemoglobin (HbA1c)

WBC Count Testing		
Range	Interpretations	Scoring
<4000 cells/microlitre	Lowest	0
4,500-11000 cells/microlitre	Normal	0.5
Above 11000 with every 1000cells/mm3	Diabeties risk inrease by 75%	1

d. WBC Count Testing

Blood Glucose Range	Interpretations	Scoring
<70mg/dl	Hypoglycaemia	0
84-150mg/dl	Normoglycemia	0.5
250-400mg/dl	Hyperglycaemia	1

e. Fasting Blood Glucose Testing

Range Kg/m2	Significance	Score
18.5-24.9 kg/m2	Healthy	0
≤ 25.0	Overweight	0.5
≥30kg/m2	Lifetime increased risk of diabetes	1

e. BMI

C. Clinical parameters of obesity associated with cancer/prostate cancer

Clinical Significances of a Gene	Binary Scoring
Uncertain Significance	-1
Likely Benign	-0.5
Benign	0
Likely Pathogenic	0.5
Pathogenic	1

a. Clinical Significance

Clinical Significances of a Gene	Frequency Threshold values	Binary Score
Uncertain Significance	-	-
Likely Benign	-	-
Benign	-	-
Likely Pathogenic	>0.05	0
Pathogenic	<0.05	1

b. Minor allele frequency

Clinical Significances of a Gene	Range Kg/m2	Significance	Score
Uncertain Significance	-	-	-
Likely Benign	-	-	-
Benign	18.5-24.9 kg/m2	Healthy	0
Likely Pathogenic	≤ 25.0	Overweight	0.5
Pathogenic	≥30kg/m2	Lifetime increased risk of diabetes	1

e. BMI

FAT type	Threshold Levels	Obesity Risk	PCa Risk	Scoring
Total Cholesterol	125 to 200mg/dL	-	-	-
Non-HDL	Less than 130mg/dL	High risk of obesity	Increased risk of cancer	0.5
LDL	Less than 100mg/dL	Obesity	Increased risk of cancer	1
HDL	40mg/dL or higher	Non-obese	Lower risk of cancer	0

d. HDL/LDL

Supplementary Fig. (1).

OVERALL SURVIVAL

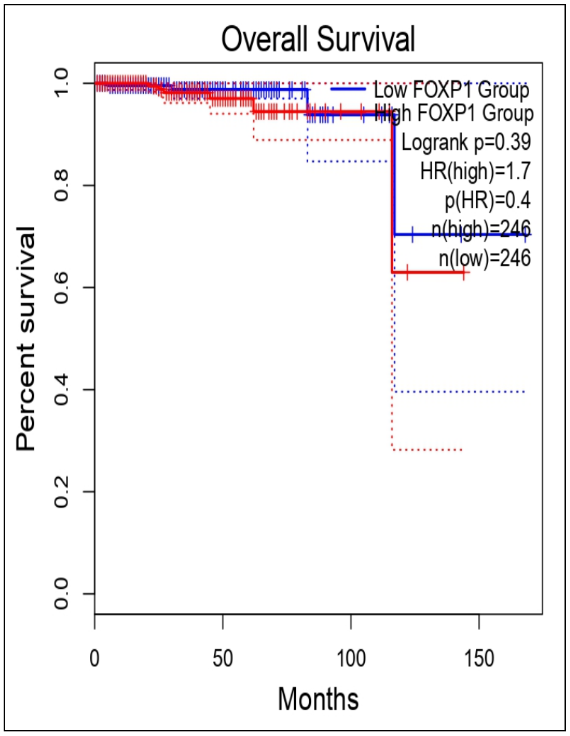

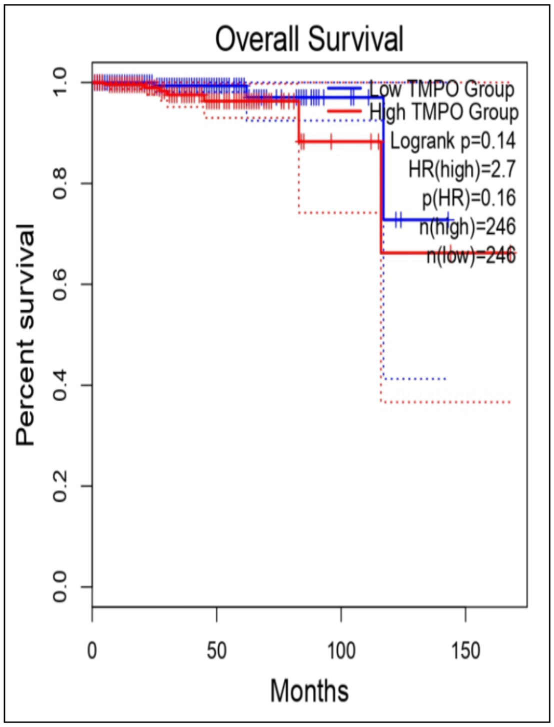

OVERALL SURVIVAL

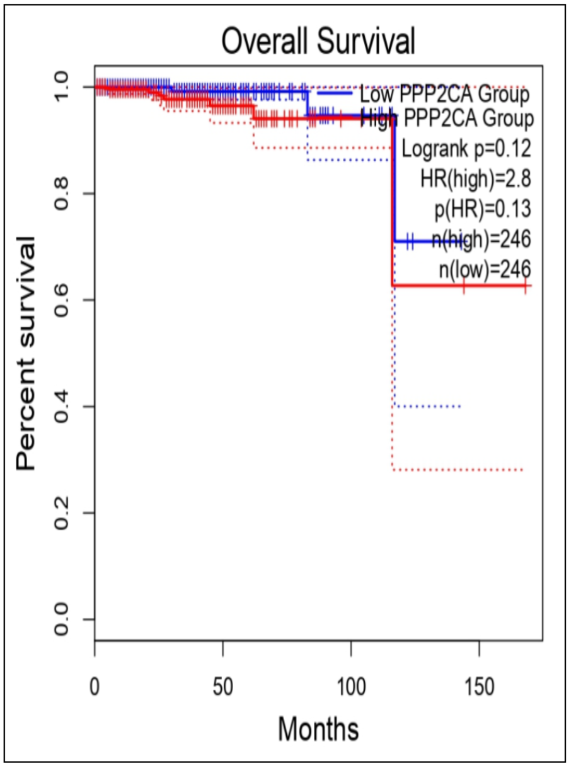

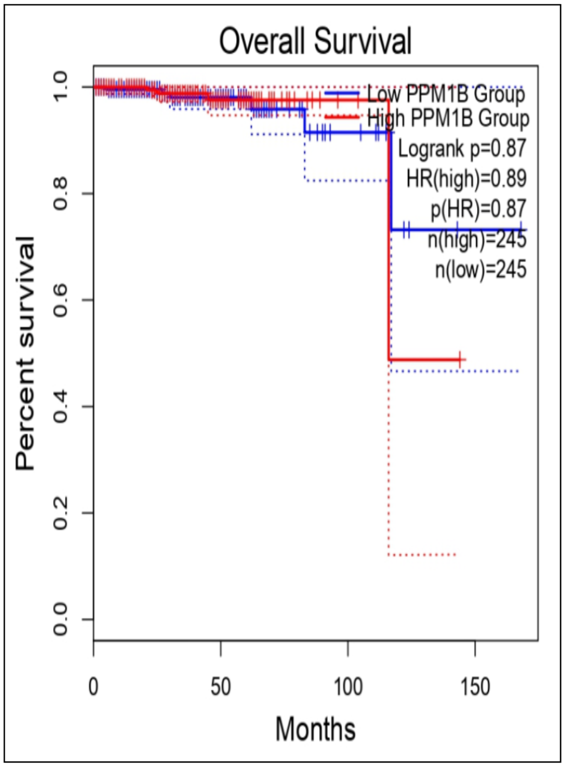

OVERALL SURVIVAL

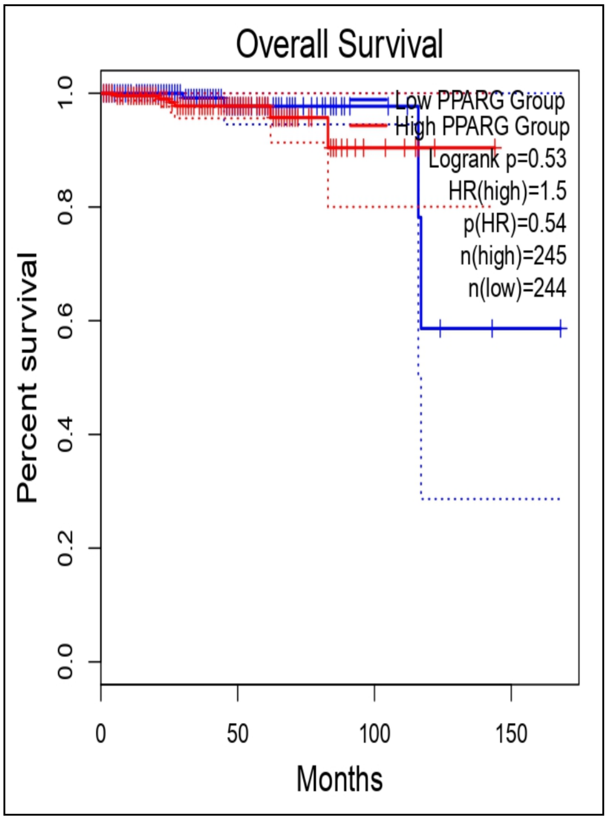

OVERALL SURVIVAL

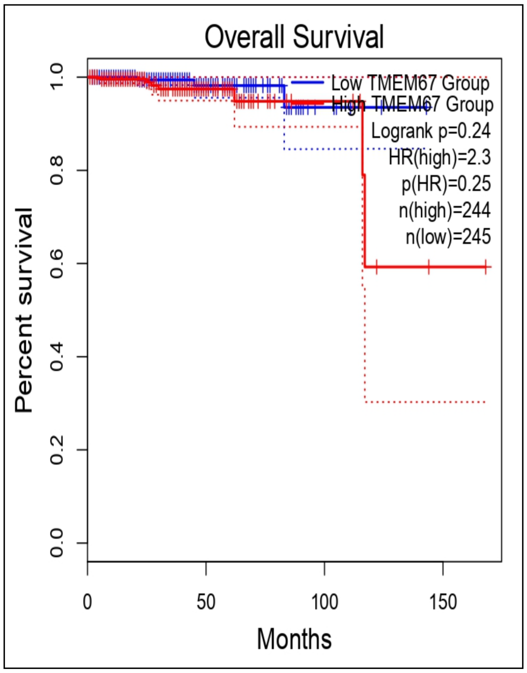

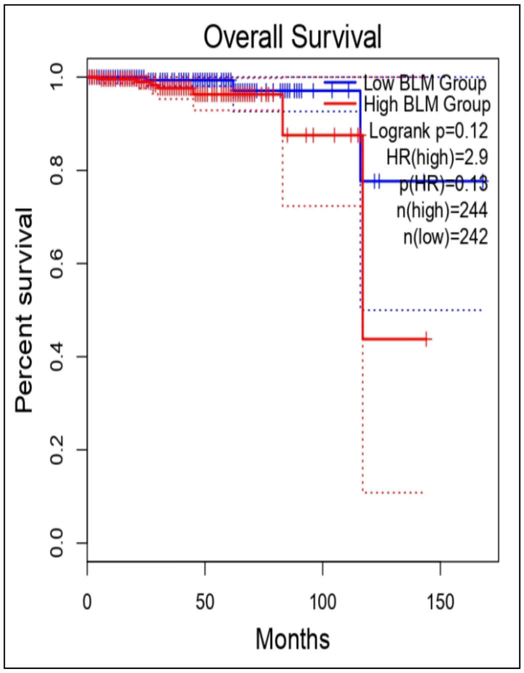

DISEASE FREE SURVIVAL

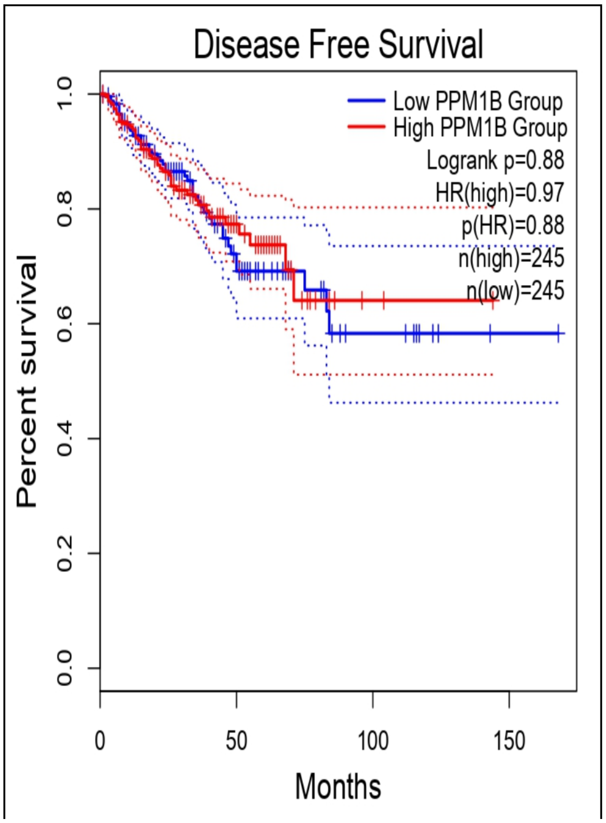

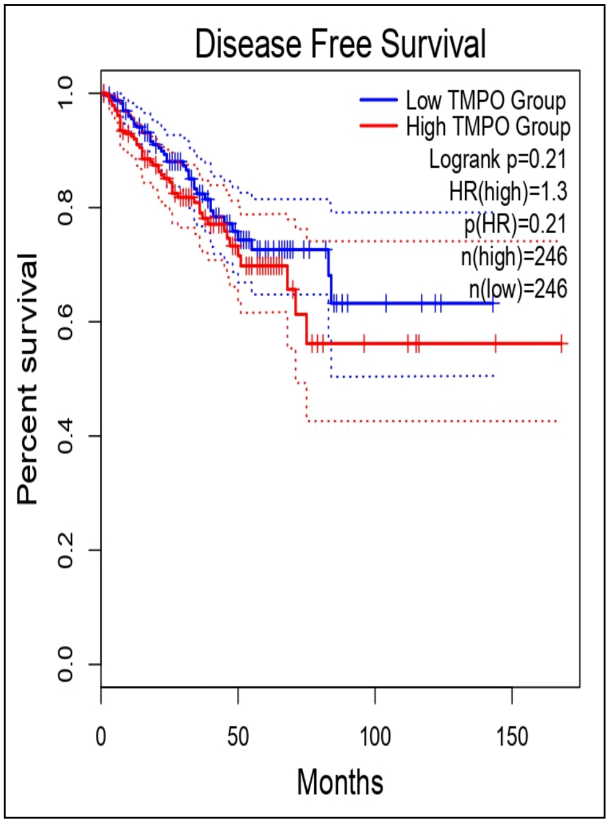

DISEASE FREE SURVIVAL

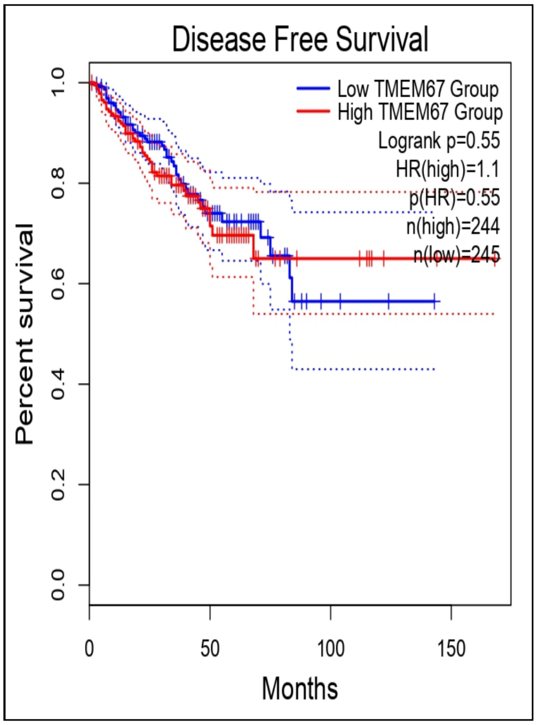

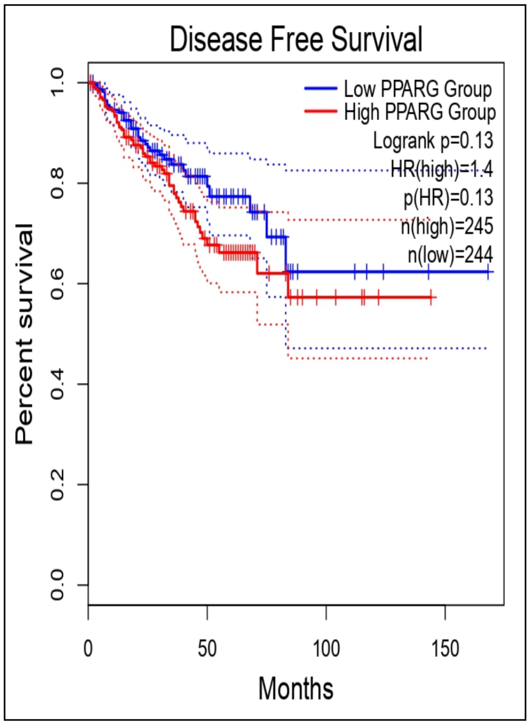

DISEASE FREE SURVIVAL

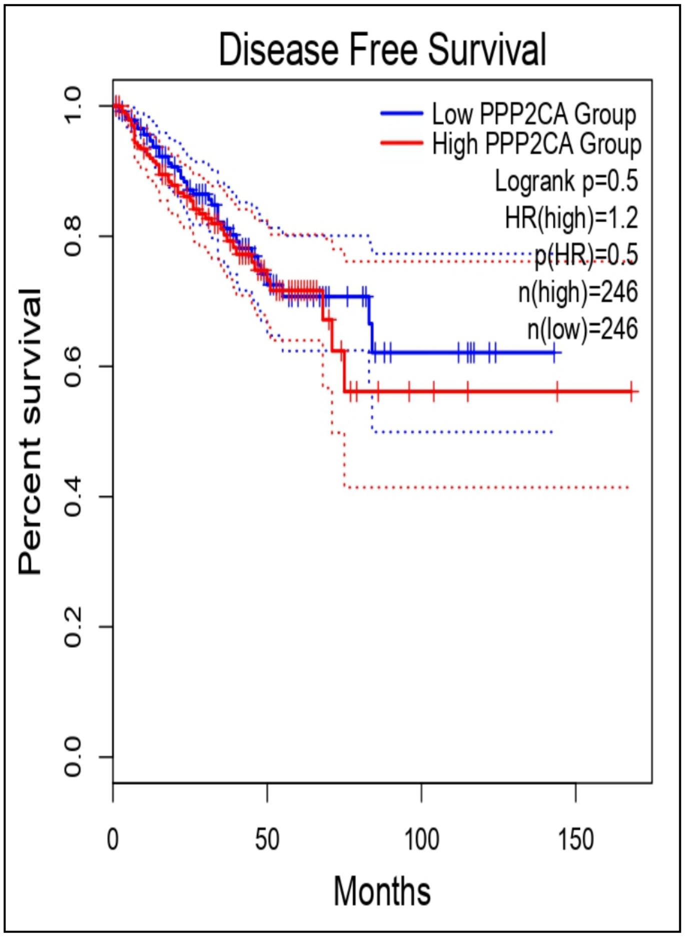

Supplementary Fig. (2).
